# Electronic Structures of Penta-SiC_2_ and g-SiC_3_ Nanoribbons: A First-Principles Study

**DOI:** 10.3390/ma16114041

**Published:** 2023-05-29

**Authors:** Zhichao Liu, Xiaobiao Liu, Junru Wang

**Affiliations:** 1 School of Physics and Electronic Informations, Yantai University, Yantai 264005, China; 2 School of Sciences, Henan Agricultural University, Zhengzhou 450002, China

**Keywords:** electronic structures, nanoribbons, silicon–carbon compounds, Li-ion batteries, first principles

## Abstract

The dimensions of nanoribbons have a significant impact on their material properties. In the fields of optoelectronics and spintronics, one-dimensional nanoribbons exhibit distinct advantages due to their low-dimensional and quantum restrictions. Novel structures can be formed by combining silicon and carbon at different stoichiometric ratios. Using density functional theory, we thoroughly explored the electronic structure properties of two kinds of silicon–carbon nanoribbons (penta-SiC_2_ and g-SiC_3_ nanoribbons) with different widths and edge conditions. Our study reveals that the electronic properties of penta-SiC_2_ and g-SiC_3_ nanoribbons are closely related to their width and orientation. Specifically, one type of penta-SiC_2_ nanoribbons exhibits antiferromagnetic semiconductor characteristics, two types of penta-SiC_2_ nanoribbons have moderate band gaps, and the band gap of armchair g-SiC_3_ nanoribbons oscillates in three dimensions with the width of the nanoribbon. Notably, zigzag g-SiC_3_ nanoribbons exhibit excellent conductivity, high theoretical capacity (1421 mA h g^−1^), moderate open circuit voltage (0.27 V), and low diffusion barriers (0.09 eV), making them a promising candidate for high storage capacity electrode material in lithium-ion batteries. Our analysis provides a theoretical basis for exploring the potential of these nanoribbons in electronic and optoelectronic devices as well as high-performance batteries.

## 1. Introduction

Since the discovery of graphene in 2004, the two-dimensional (2D) layered structure has attracted significant scientific interest [1]. Graphene is a 2D crystalline material composed entirely of carbon atoms with sp^2^ hybridization orbitals. The pz orbitals perpendicular to the graphene plane form energy bands with linear dispersion relations near the Fermi level, exhibiting the characteristics of a Dirac cone, which endows graphene with excellent electrical and thermal conductivity properties [2,3]. Due to the robust bond framework formed by sp^2^ hybridization in carbon atoms, graphene is currently the most mechanically strong 2D material [4]. From the 2D graphene, numerous other materials, such as zero-dimensional fullerenes, one-dimensional carbon nanotubes, and carbon nanoribbons, can be derived. It is worth noting that carbon has four outer electrons that can form three different hybridization states: sp^2^ (graphene), sp^3^ (diamond), and sp (ethylene). Although silicon and carbon are in the same column of the periodic table, their bonding characteristics are markedly different. Silicene, a 2D honeycomb lattice made up of sp^2^-sp^3^ hybridization, possesses several superior properties and good compatibility in devices that make it a promising substitute for graphene. Currently, silicon is the main component of semiconductor electronic products. However, both graphene and silicene exhibit zero band gaps, which limits their widespread use in optoelectronic devices such as LEDs, field-effect transistors, and solar cells. Searching for new 2D materials that surpass traditional materials will have significant implications for future applications. For instance, combining silicon and carbon to form 2D binary compounds could lead to novel material properties. Different stoichiometric ratios of carbon and silicon generate novel 2D layered structures, such as SiC [5], folded pentagonal SiC_2_ (penta-SiC_2_) [6], SiC_2_ siligraphene (g-SiC_2_) [7], SiC_2_ silagraphene [8], SiC_3_ siligraphene (g-SiC_3_) [9], and SiC_7_ siligraphene (g-SiC_7_) [10]. These silicon–carbon 2D materials have exhibited excellent performances in lithium-ion batteries, electrocatalysis, and optoelectronic fields [11,12,13,14]. For silicon–carbon compounds, studying the 2D layered isomers of silicon–carbon with medium energy gaps (1.0–2.0 eV) is an interesting issue that has attracted significant attention in the scientific community.

The properties of materials are closely related to their dimensions. One-dimensional (1D) nanostructures, such as nanoribbons, nanotubes, and nanowires, display properties beyond those of 2D systems because of their low dimensionality and quantum confinement. They exhibit electronic bandgap changes associated with quantum confinement and edge effects, as well as local spin-polarized edge states with specific edge geometries. This makes 1D nanostructures promising candidates for spintronics, optoelectronics, and other applications that require tailored electronic and magnetic properties and has attracted widespread attention over the past two decades. For example, graphene nanoribbons (GNRs) exhibit unique properties that are distinct from those of bulk graphene when cut into smaller widths [15,16,17]. Zigzag graphene nanoribbons (ZGNRs) have been predicted to be antiferromagnetic semiconductors [18], and their electronic and magnetic properties can be tuned by doping or introducing defects [19,20,21]. Recently, Professor Song Jiang’s team published a report in *Science* about the topological localized excitons found in single armchair graphene nanoribbons. The findings of their research offer a nanoscale solution for schemes that incorporate the electron, magnetic, and photon degrees of freedom in quantum mechanics [22]. Different edge states of 1D nanoribbons generally have distinct properties. Inspired by the extensive research on GNRs for their electronic properties and the lack of theoretical studies on penta-SiC_2_ and g-SiC_3_ nanoribbons, we explored the electronic structures of penta-SiC_2_ and g-SiC_3_ nanoribbons. Considering the electrical conductivity of zigzag g-SiC_3_ nanoribbons, we explore the potential of zigzag g-SiC_3_ nanoribbons as anode electrode materials for LISs. Similar materials have been reported experimentally, such as the application of Si@SiC composite and graphene nanoribbons in the field of lithium-ion batteries [23,24,25]. Furthermore, there have been theoretical reports on nanoribbons [26]. A comprehensive investigation into the variations in structure and performance of silicon carbide nanoribbons can potentially clarify their edge electronic spin states and promote their applications in optoelectronics, spintronics, and batteries.

Using first-principles calculations, we investigated the electronic properties of two types of silicon carbide nanoribbons, namely penta-SiC_2_ and g-SiC_3_ nanoribbons, with varying widths, growth directions, and edge environments. The results revealed that the electronic properties of these nanoribbons were strongly influenced by their orientation and width. Specifically, the a-type penta-SiC_2_ nanoribbon exhibited characteristics of an antiferromagnetic semiconductor, which could be beneficial for the development of next-generation optoelectronic devices. Meanwhile, the bandgap of armchair g-SiC_3_ nanoribbons exhibited a 3D oscillation pattern with increasing width. It is worth noting that zigzag g-SiC_3_ nanoribbons show promise as a potential electrode material for lithium-ion batteries because of their excellent conductivity, a theoretical capacity of 1421 mA h g^−1^, the moderate open circuit voltage, and low diffusion barriers. By systematically studying the electronic properties of these nanoribbons with different widths and orientations, we can identify the desired material properties and promote the application of such materials in the field of optoelectronics and batteries.

## 2. Materials and Methods

This study employs first-principles calculations based on density functional theory using the VASP (Vienna ab initio simulation) software package to calculate relevant geometric structures, electronic structures, and energies [27]. The electron–ion interactions were described using the projector-augmented-wave (PAW) potentials [28]. The exchange-correlation functional is based on the generalized gradient approximation (GGA) in the Perdew–Burke–Ernzerhof (PBE) form [29,30]. To verify the results of the PBE calculations, the electronic structures of some penta-SiC_2_ and g-SiC_3_ nanoribbons were calculated using the hybrid Heyd–Scuseria–Ernzerhof (HSE06) functional [31]. Plane waves with a cutoff energy of 520 eV are applied to the Kohn–Sham electronic wave function, and we optimize the structures using the conjugate gradient (CG) method until the maximum force on each atom is less than 0.01 eV/Å. To avoid interactions with neighboring phases, the nanoribbons are repeatedly arranged along the *x^-^* direction, while a 15 Å vacuum layer is applied in the *y*^−^ and *z*^−^ directions. The Brillouin zone was sampled using a 15×15×1 k-point grid for 2D penta-SiC_2_ and g-SiC_3_, while for the nanoribbon model, a 11×1×1 grid was adopted [32]. We consider spin polarization in all calculations, assigning different spin configurations to obtain the ground-state magnetic configuration. To evaluate the stability of the material, we determine the binding energy (*E_b_*) using the formula $E_{b}=(xE_{Si}+yE_{c}-E_{Si_{x}C_{y}})/(x+y)$, where $E_{\mathrm{Si}}$, $E_{C}$, and $E_{{\mathrm{Si}}_{x}C_{y}}$ represent the energy of Si atoms, C atoms, and the nanostructured material, respectively. The energetic minimal-path profiles for the lithium diffusion in nanoribbons were determined by using the climbing image nudged elastic band (CNEB) method as implemented in the VASP transition state tools [33,34]. The structures were relaxed during the CNEB processes.

## 3. Results and Discussion

### 3.1. Electronic Structures of 2D Penta-SiC_2_/g-SiC_3_

**Figure 1:**
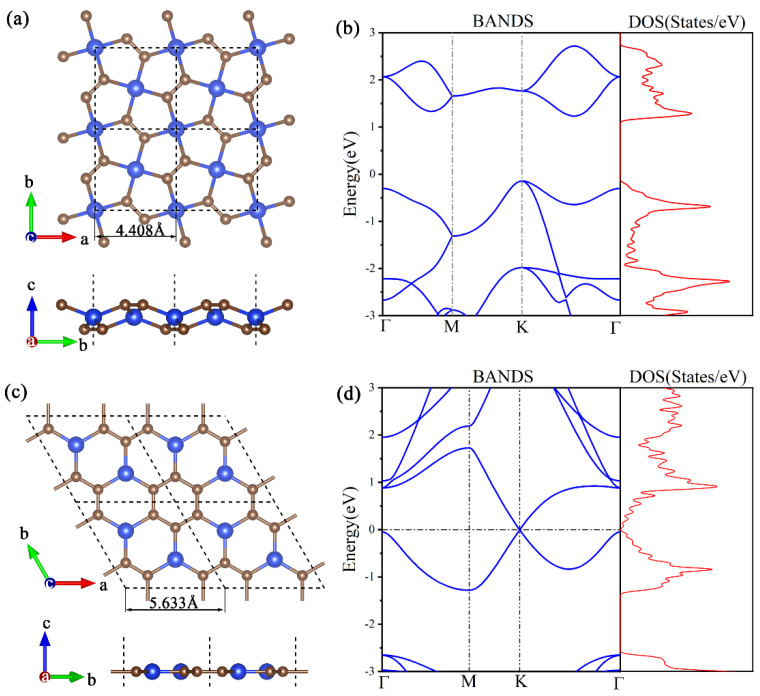
(**a**) Crystal structure of 2D penta−SiC_2_. It shows a 2 × 2 supercell, with Si depicted by blue spheres and C depicted by grey spheres. (**b**) (left panel) Electronic band structure and (right panel) electron density of states (DOS) of the 2D penta−SiC_2_ crystal. (**c**) Crystal structure of 2D g−SiC_3_. (**d**) (left panel) Electronic band structure and (right panel) DOS of the 2D g−SiC_3_.

Figure 1a illustrates the folded structure of penta-SiC_2_, where each unit cell consists of two Si atoms and four C atoms. The optimized Si-C bond length is 1.908 Å, and the C-C bond length is 1.362 Å. According to the electronic band structure in Figure 1b, penta-SiC_2_ is an indirect bandgap semiconductor with a bandgap of 1.38 eV and 2.49 eV at PBE and HSE06 levels, respectively. The results agreed well with the previous literature [6]. Moreover, the Bader analysis suggests that electrons transfer from Si to C. In addition, the binding energy of penta-SiC_2_ is 7.297 eV/atom, which is lower than that of graphene (9.176 eV/atom) but higher than that of g-SiC_2_ siligraphene (6.46 eV/atom) [7].

The g-SiC_3_ siligraphene belongs to the hexagonal space group P6/mmm, as shown in Figure 1c. It is a planar structure with no wrinkles, with two Si and six C atoms in the primitive cell. Hexagonal six-carbon rings are formed by each C atom bonding with one Si and two C atoms, while each Si atom bonds with three C atoms. The Si-C bond length measures 1.813 Å, while the C–C bond length is 1.439 Å. The results agreed well with the previous literature [9,35]. The g-SiC_3_ is a hybrid of Si and C atoms, exhibiting characteristics similar to graphene. The C–C bond (1.439 Å) shows the characteristic of weak C=C double bonding because it is slightly longer than that of graphene (1.42 Å) but shorter than the C–C single bond in ethane (1.54 Å). The Bader analysis shows that electrons are transferred from Si to C. The electronic band structure (Figure 1d) reveals that g-SiC_3_ is a semimetal. The binding energy of g-SiC_3_ is 7.795 eV/atom, which is lower than that of graphene (9.176 eV/atom) but higher than that of g-SiC_2_ siligraphene (6.46 eV/atom) [7]. Unlike graphene with pure sp^2^ hybridization, Si atoms prefer to adopt sp^2^-sp^3^ hybridization and form the buckled structure of silicene, making the stability of planar silicene decrease as the number of Si atoms increases.

This study investigates the electronic structural characteristics of penta-SiC_2_ and g-SiC_3_ nanoribbons, taking into account their unique properties as one-dimensional nanostructures. Due to their low dimensions and quantum confinement effects, nanoribbons demonstrate superior functionality when compared to 2D systems. Specifically, nanoribbons exhibit modifications in electronic bandgaps associated with quantum confinement and edge effects, as well as local spin-polarized edge states specific to the edge geometry. Considering the aforementioned qualities, we seek to better understand the electronic structure of penta-SiC_2_ and g-SiC_3_ nanoribbons.

### 3.2. Electronic Structures of Penta-SiC_2_ Nanoribbons

**Figure 2:**
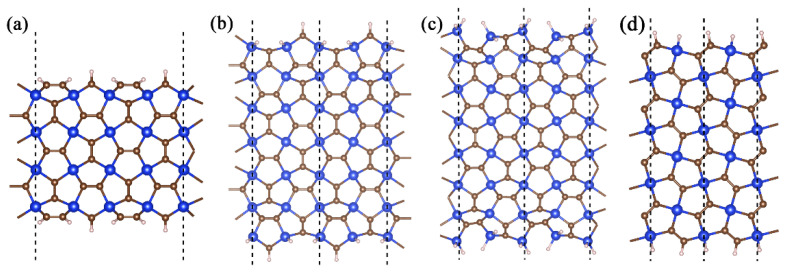
Structures of four types of penta-SiC2 nanoribbons. (**a**) a−SiC2 nanoribbon is made up of carbon atoms located at the edges. (**b**) b−SiC2 nanoribbon has edge atoms composed of Si and C. (**c**) c−SiC2 nanoribbon has edge silicon atoms that are saturated with two hydrogen atoms. (**d**) d−SiC2 nanoribbon differs from the previous three types in direction, which are composed of silicon and carbon atoms that have been saturated with hydrogen atoms.

The electronic properties of penta-SiC_2_ nanoribbons were analyzed to gain insight into their behavior under different conditions first. Four types of nanoribbon structures were examined while taking into account different directions and end atoms. The nanoribbon’s width was defined as the number of edge silicon atoms with each edge saturated with hydrogen atoms. The penta-SiC_2_ nanoribbons maintained their folding characteristics. In the subsequent sections, we will discuss the properties of these four types of nanoribbons in the order presented in Figure 2. Through these analyses, we aim to gain a more comprehensive understanding of the behavior of penta-SiC_2_ nanoribbons in different conditions.

**Figure 3:**
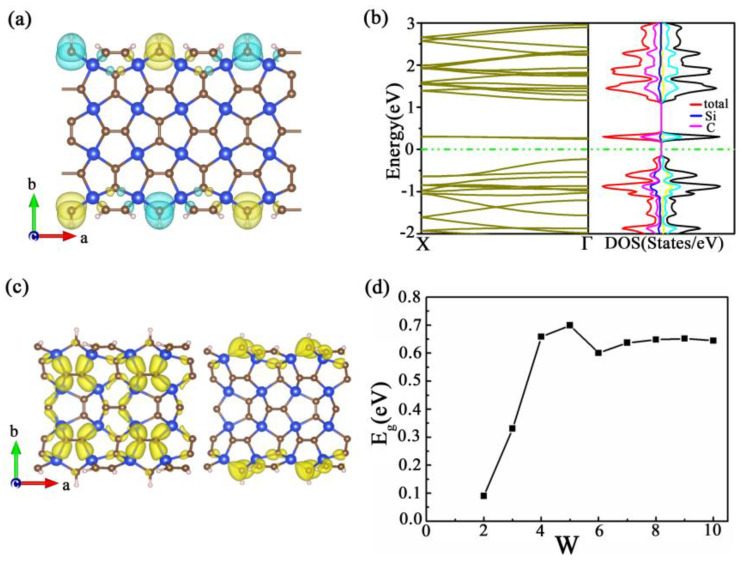
(**a**) The spin-polarized electron density for AFM orderings. (**b**) Electronic band structure and electron density of states of penta−SiC_2_ nanoribbons. (**c**) Partial−charge density distributions of the valence band and conduction band of Г. (**d**) The variation of the band gap of nanoribbons with width.

The first type of nanoribbons, called a-SiC_2_, are made up of carbon atoms located at the edges. We investigated the spin configuration of these nanoribbons and found that the antiferromagnetic configuration (AFM) is the most stable, with magnetic moments mainly concentrated on the edge carbon atoms, as shown in Figure 3. This electron structure property can be observed in nanoribbons of different lengths. Taking a nanoribbon with a width of 4 (w = 4) as an example, we observe a direct bandgap at the Γ point in the electron band structure, indicating the properties of a direct bandgap semiconductor, and the band gaps are 0.66 eV and 1.60 eV at PBE and HSE06 levels, respectively. Upon assigning different spin configurations, the magnetic moment was found to be concentrated near the edge carbon atoms, with adjacent spins arranged in opposite directions. The antiferromagnetic coupling of edge atoms is more stable. The energy difference between the antiferromagnetic coupling and the absence of spin polarization is 0.227 eV/atom, slightly lower than that of GNRs (0.3 eV). Moreover, as shown in Figure 3, the highest occupied state at point Г is contributed by the p_y_ orbital of carbon, while the lowest unoccupied state at points Г is contributed by the p_z_ orbital of the edge carbon. The bandgap of nanoribbons increases with the increase in the nanoribbon’s width. These characteristics of the nanoribbon can be useful for the development of a new generation of optoelectronic devices.

**Figure 4:**
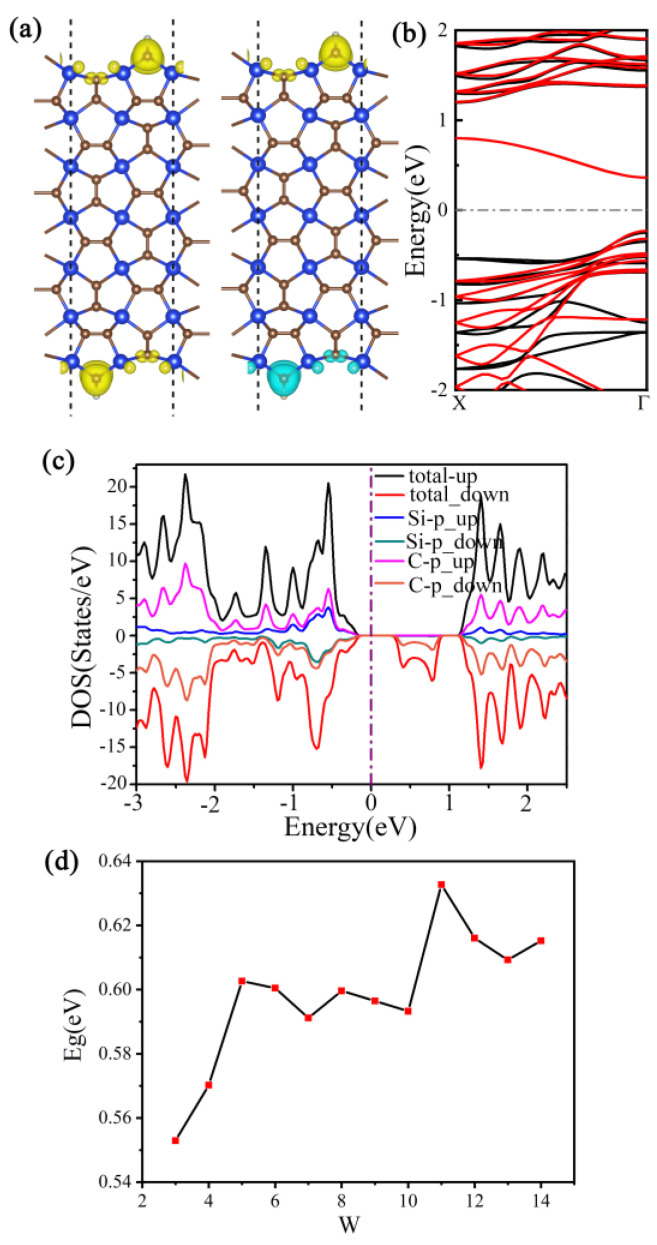
(**a**) The spin−polarized electron density for FM orderings and AFM orderings. (**b**) Electronic band structure and (**c**) electron density of state of penta-SiC_2_ nanoribbons. The energy at the Fermi level was set to zero. (**d**) The variation of the band gap of nanoribbons with width.

The second type of nanoribbon, called b-SiC_2_, has edge atoms composed of Si and C. Our research has focused on investigating the possible spin configurations of the second type of nanoribbons and we have obtained two configurations (see Figure 4a). We have found that the ferromagnetic (FM) and AFM configurations have equivalent energies, indicating that the second nanoribbon type exhibits paramagnetism. As shown in Figure 4b, the types of nanoribbons all exhibit semiconductor properties. For example, when the nanoribbon width is 7 (w = 7) and the AFM arrangement is used, the spin-up and spin-down orbitals overlap, forming a direct bandgap semiconductor at the point Г. The band gaps are 0.59 eV and 1.84 eV at the PBE and HSE06 levels, respectively. In this case, the conduction band minimum (CBM) is determined by the C p-orbital, while the valence band maximum (VBM) is collectively contributed by the C and Si p-orbitals (see Supplementary Figure S1). Conversely, when using the FM arrangement, the spin-up and spin-down orbitals split, and we can observe that the size of the bandgap will be determined mainly by the spin-down orbitals, resulting in another direct bandgap semiconductor at the point Г (Figure 4b). The CBM remains dominated by the C p-orbital, while the VBM is shared by the C and Si p-orbitals (Figure 4c). As shown in Figure 4d, the bandgap of the spin-down orbitals exhibits minor three-dimensional oscillations with increasing nanoribbon width starting from w = 5. On the other hand, the bandgap of the spin-up orbitals decreases with the increasing nanoribbon width (see Supplementary Figure S1).

**Figure 5:**
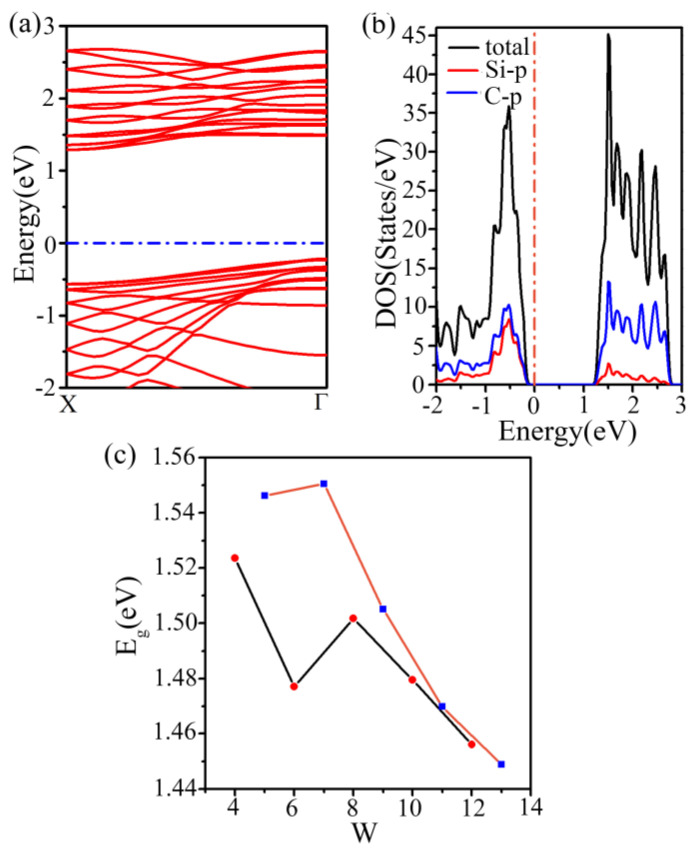
(**a**) Electronic band structure and (**b**) electron density of state of the third class of penta−SiC_2_ nanoribbons (w = 9). (**c**) The variation of the band gap of nanoribbons with width w.

The third nanoribbon type, called c-SiC_2_, has edge silicon atoms that are saturated with two hydrogen atoms and possess the same orientation as the first type. It is a nonmagnetic semiconductor with an indirect bandgap (Figure 5a). The band gaps (w = 9) are 1.51 eV and 2.51 eV at the PBE and HSE06 levels, respectively. In this study, we present the band diagram for a nanoribbon with a bandwidth of nine (w = 9), noting that the band structures for the other widths are similar. From the electron density of states (DOS) shown in Figure 5b, we can find that valence bands and conduction bands near the Fermi level are mostly dominated by the p orbitals of C and Si. To categorize this type of nanoribbon, we divided them into two categories based on the surrounding of terminating atoms and generated a bandgap variation diagram with different widths. We found out the bandgap decreases with the increasing width of the nanoribbon.

**Figure 6:**
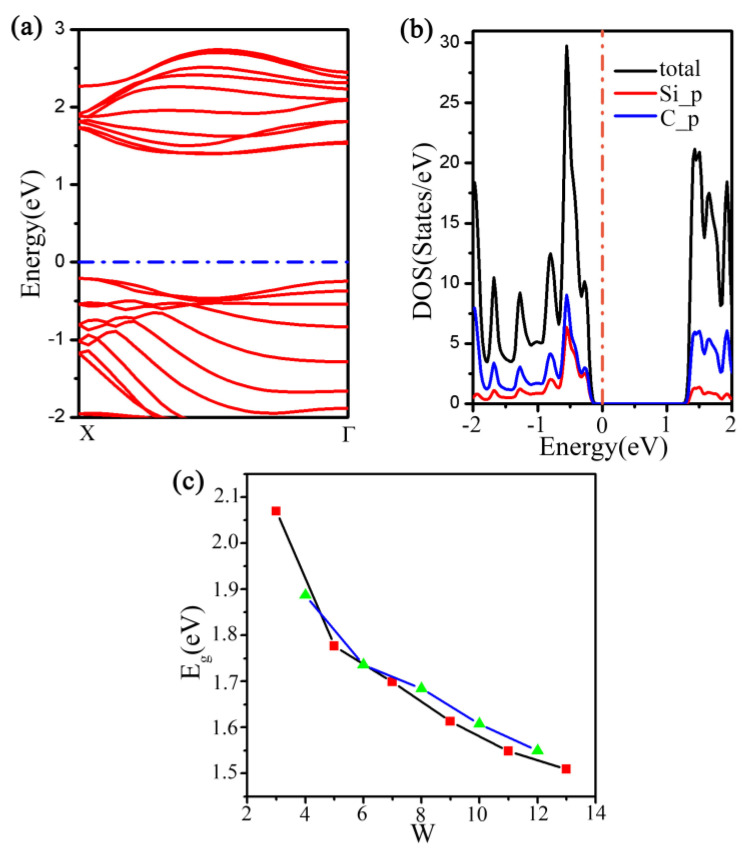
(**a**) Electronic band structure and (**b**) electron density of state of the fourth class of penta−SiC_2_ nanoribbons (w = 10). (**c**) The variation of the band gap of nanoribbons with width.

The fourth type of nanoribbon (d-SiC_2_) differs from the previous three types in direction, which are composed of silicon and carbon atoms that have been saturated with hydrogen atoms. In this study, we provide a comprehensive analysis of the band structure and density of states for a nanoribbon with a width of 10 (w = 10). This type of nanoribbon is a nonmagnetic indirect bandgap semiconductor (Figure 6a). The band gaps are 1.61 eV and 2.63 eV at the PBE and HSE06 levels, respectively. Our findings reveal that the p orbitals of silicon and carbon contribute to both the lowest unoccupied and highest occupied bands, with carbon’s p orbitals being more prominent in the lowest unoccupied energy level. Notably, the width of the nanoribbon significantly affects its bandgap, with the bandgap decreasing correspondingly with an increase in width. It is worth noting that the appropriate width range of nanoribbons can provide an intermediate bandgap material, which could be useful in applications such as field-effect transistors and solar cells.

### 3.3. Electronic Structures and Applications of g-SiC_3_ Nanoribbons

**Figure 7:**
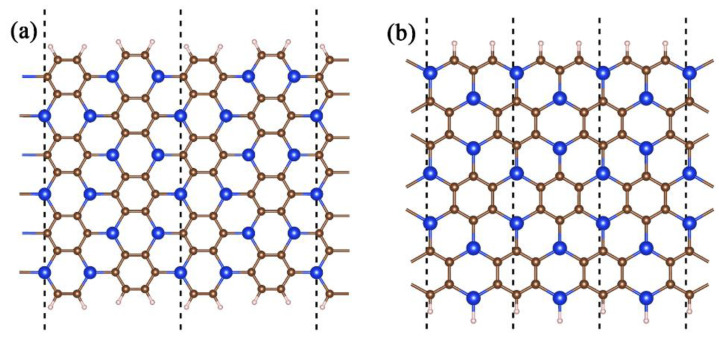
Structure of (**a**) armchair type (w = 13) and (**b**) zigzag type (w = 7) of g-SiC_3_ nanoribbons.

We explored the electronic properties of g-SiC_3_ siligraphene nanoribbon by examining two kinds of nanoribbon structures: zigzag and armchair. For the armchair nanoribbon, we established the nanoribbon width as the number of atoms in the direction of the ribbon, whereas for the zigzag nanoribbon, it was defined as the number of atoms along the zigzag chain. After optimizing the structure as depicted in Figure 7, we saturated all edge dangling bonds with hydrogen atoms, and the nanoribbon atoms remained on the same plane, with some slight changes in bond lengths. We subsequently analyzed the characteristics of each nanoribbon type. In summary, through the use of both types of nanoribbons, we thoroughly investigated the electronic properties of g-SiC_3_ siligraphene nanoribbons and theoretical study on zigzag g-SiC_3_ nanoribbons as electrode materials for lithium-ion batteries (LIBs).

#### 3.3.1. Electronic Structures of Armchair g-SiC_3_ Nanoribbons

**Figure 8:**
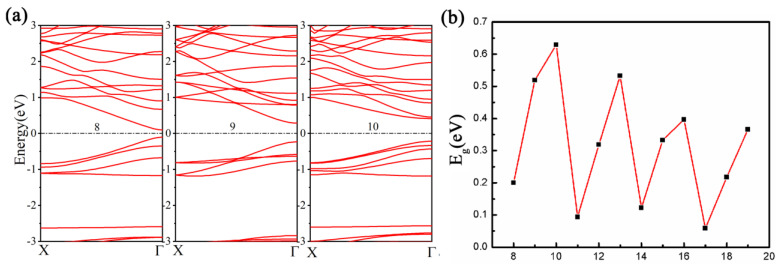
(**a**) Electronic band structure of armchair g−SiC_3_ nanoribbons when w = 8, 9, and 10. (**b**) The variation of the band gap of armchair nanoribbons with width.

The calculations have shown that this nanoribbon is a direct bandgap semiconductor with the gap located at Г point from the energy-band structure in Figure 8a, which is consistently similar regardless of the nanoribbon length. Additionally, when w = 8, 9, and 10, the PBE band gaps were 0.20 eV, 0.52 eV, and 0.63 eV, and the HSE06 band gaps were found to be 0.30 eV, 0.73 eV, and 0.89 eV, respectively. More notably, Figure 8b shows that the nanoribbon’s bandgap undergoes a clear three-dimensional oscillation with changes in the nanoribbon width; the overall magnitude of the bandgap tends to decrease as the width of the nanoribbon increases. To gain further insight into the electronic properties of the nanoribbon, we conducted an indepth analysis of a 13-atom-wide nanoribbon. Both the highest occupied and lowest unoccupied states predominantly arise from Si and C atoms. Our Bader analysis indicates that Si loses approximately 2.374 electrons, while H loses only 0.056 electrons. In contrast, edge C atoms gain around 0.028 electrons, with the remaining C atoms gaining about 0.779 electrons, as seen in the differential density of states plot (see Supplementary Figure S2). Specifically, the charge density plot at the Г point shows that Si-C bonding dominates the highest occupied state, while the lowest unoccupied state exhibits significant contributions from both Si–C and C–C bonding (see Supplementary Figure S2).

#### 3.3.2. Electronic Structures of Zigzag g-SiC_3_ Nanoribbons

**Figure 9:**
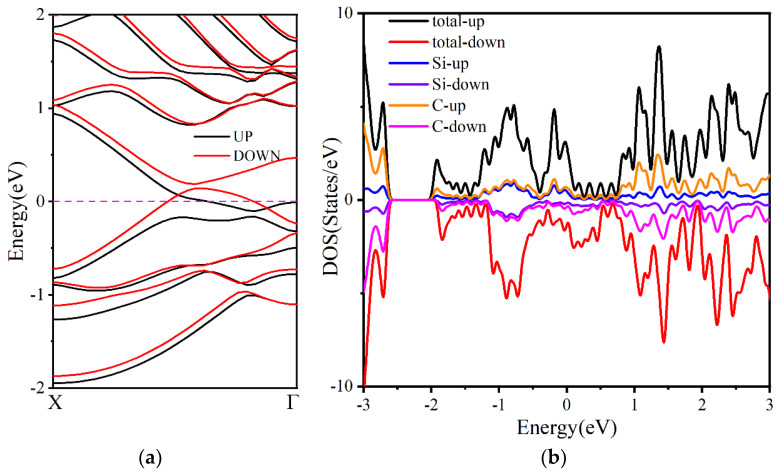
(**a**) Electronic band structure and (**b**) electron density of state of zigzag g−SiC_3_ nanoribbons when w = 7.

We investigated the electronic structure and potential of zigzag g-SiC_3_ nanoribbons with a width of seven (w = 7) as anode electrode materials for LISs. Our analysis reveals that nanoribbons of different widths exhibit metallic behavior. The electronic band structure for both PBE and HSE06 methods of the w = 7 nanoribbon shows that both spin-up and spin-down orbitals cross the Fermi level, indicating metallic behavior (Figure 9a). In addition, the electronic density of the states reveals that the states near the Fermi level are mainly contributed by Si and C atoms (Figure 9b). Furthermore, the edge atoms of the nanoribbon possess a local magnetic moment, and the top and bottom atoms exhibit the same direction, leading to ferromagnetic behavior with a total magnetic moment of 0.59 $\mu_{Β}$ (see Supplementary Figure S3). Finally, we observe an energy difference of 31.2 meV between spin-polarized ferromagnetism and nonspin-polarized states.

#### 3.3.3. Zigzag g-SiC_3_ Nanoribbon: A Potential Anode Material for LIBs

**Figure 10:**
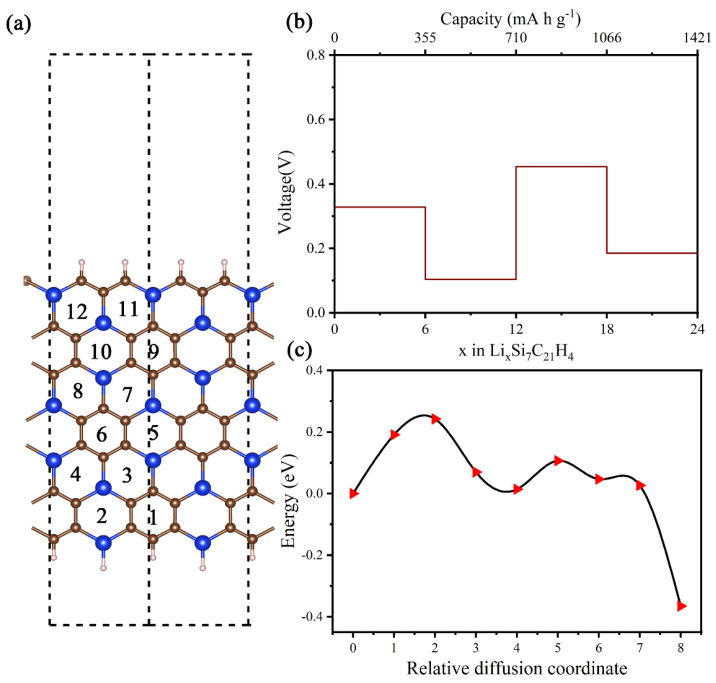
(**a**) Atomic structure of the zigzag g−SiC_3_ nanoribbons, with labeled inset adsorption sites. (**b**) Voltage profiles of g-SiC_3_ nanoribbons at different Li concentrations. (**c**) Energy profile for Li diffusion in the g-SiC_3_ nanoribbons along the path 6→4→1.

Zigzag nanoribbons possess excellent conductivity, making them a viable option as electrode materials for lithium-ion batteries. Our investigation begins by examining possible adsorption sites of lithium on the nanoribbons. By performing calculations, we find that Li stabilizes in the central position of a hexagon (H site). Figure 10 provides visual representations of the 12 potential adsorption sites, with position 1 proving the most stable. The Li atom placed at the top site of Si/C atoms moves to the H site during structural. The formation energy of Li adsorption on the nanoribbon can be defined as

$$E_{\mathrm{form}}=(E_{\mathrm{Li}+\mathrm{nanoribbon}}-E_{\mathrm{nanoribbon}}-E_{\mathrm{Li}}),$$

where ***E****_Li+nanoribbon_* and ***E****_nanoribbon_* represent the total energies of zigzag g-SiC_3_ nanoribbons with and without Li adsorption and ***E****_Li_* is the energy of Li in the bulk crystal. The formation energies for each adsorption site are listed in Table 1. The table indicates that the closer Li is to the edge of the nanoribbon, the lower its formation energy. Negative formation energies imply that Li atoms have a preference for adsorbing on zigzag g-SiC_3_ nanoribbons as opposed to forming Li clusters.

The performance of LIBs is affected by two important parameters: the average electrode potential and the *Li* storage capacity. To evaluate the average open circuit voltage (*V_ave_*), we consider a typical half-cell reaction against Li/Li^+^: ${\mathrm{Li}}_{1-x}Si_{7}C_{21}H_{4}+x{\mathrm{Li}}^{+}+xe^{-}↔{\mathrm{Li}}_{x}Si_{7}C_{21}H_{4}$. Neglecting volume and entropy effects, we can estimate *V_ave_* by computing the total energy difference between Li-intercalated SiC_3_ nanoribbons with different *Li* concentrations using the definition [36]:

$$V_{\mathrm{ave}}=-\frac{E\left[Li_{x_{2}}Si_{7}C_{21}H_{4}\right]-E\left[Li_{x_{1}}Si_{7}C_{21}H_{4}\right]-(x_{2}-x_{1})E_{Li}}{(x_{2}-x_{1})e}$$

The resulting electrode potential ranges from 0.10 to 0.45 V, with an average value of 0.27 eV, as evidenced by Figure 10b. This potential range ensures the safety of the electrode as an anode material and increases the energy density of the battery.

The maximum theoretical capacities of Li atoms can be determined using $C=x_{\max}F/M_{{g-\mathrm{SiC}}_{3}\;\mathrm{nanoribbon}}$, where ***x***_max_ represents the maximum fraction of Li in g-SiC_3_ nanoribbon, *F* is the Faraday constant (26.8 A h mol^−1^), and $M_{{g-\mathrm{SiC}}_{3}\;\mathrm{nanoribbon}}$ is the atomic mass of the zigzag g-SiC_3_ nanoribbon (452.7 g mol^−1^). The theoretical capacity of g-SiC_3_ nanoribbon, estimated to be 1421 mA h g^−1^ using the aforementioned formula, is approximately 3.8 times higher than that of commercial graphite (372 mA h g^−1^) [37] and the capacity is higher than that of GeS (256 mA h g^−1^) [38], Mo_2_C (526 mA h g^−1^) [39], NbGe_2_N_4_ (547 mA h g^−1^) [40], and dumbbell silicene (1002 mA h g^−1^) [41]. Despite the growing interest in 2D anode materials in recent years, only a few have been shown to exceed theoretical capacities of 1000 mA h g^−1^. This highlights the potential of g-SiC_3_ nanoribbon as a high-capacity LIB anode material. The remarkable capacity can be ascribed to the unique arrangement of Si and C atoms. Overall, the promising features of g-SiC_3_ nanoribbon make it an attractive candidate for high-capacity LIB.

Using the CNEB strategy, we determined the energy profile of diffusion for a Li atom between two steady states. Specifically, we considered the diffusion path 6→4→1, as shown in Figure 10c. The diffusion barrier from position 6 to position 4 was determined to be 0.24 eV, while the barrier from positions 4 to 1 was only 0.09 eV. We also considered the diffusion paths 6→5, 6→8, 8→9, 9→12, taking into account the relatively stable adsorption sites. As shown in Figure S4, the diffusion barriers are 0.23 eV, 0.24 eV, 0.19 eV, and 0.22 eV along the diffusion path 6→5, 6→8, 8→9, and 9→12, respectively. Comparing the Li diffusion barriers in g-SiC_3_ nanoribbons to those in graphene (0.37 eV) [42], TiF3 (0.37 eV) [43], silicon (0.57 eV) [44], and phosphorene categories (0.76 eV) [36], we observed that they were much lower. These findings indicate that Li mobility is high in g-SiC_3_ nanoribbons, which implies high charging and discharging rates.

Based on the calculations presented in this section, we propose a high-capacity anode material for LIBs: the zigzag g-SiC_3_ nanoribbons. Our findings demonstrate that this nanoribbon offers an exceptionally high capacity (1421 mA h g^−1^), coupled with a low potential barrier (0.09 eV) and moderate average electrode potential (0.27 V), all of which together indicate that it has significant potential as an anode electrode material.

## 4. Conclusions

In this study, we utilized the first-principles method to investigate the electronic structures of four types of penta-SiC_2_ nanoribbons and two types of g-SiC_3_ nanoribbons with different widths. Our results suggest that the band structures of the nanoribbons are closely related to their widths and growth directions. Notably, type-a penta-SiC_2_ nanoribbons exhibited antiferromagnetic behavior and a-SiC_2_ and b-SiC_2_ had moderate band gaps, which make them suitable for applications in optoelectronic devices. Additionally, the armchair g-SiC_3_ nanoribbons showed three-dimensional oscillation of the band gap with width increase, whereas the zigzag g-SiC_3_ nanoribbons possessed metallic properties. It is worth highlighting that the zigzag nanoribbons hold potential as high-capacity anode electrode materials for LISs, displaying a high capacity of 1421 mA h g^−1^, a moderate open-circuit voltage of 0.27 V, and a low diffusion barrier of 0.09 eV. Our systematic investigation of the electronic structures of penta-SiC_2_ and g-SiC_3_ nanoribbons with varying widths and directions could contribute to finding the desired material properties needed to advance their applications in fields such as optoelectronics and battery technology.

## Figures and Tables

**Table 1:** Calculated the formation energies for each adsorption site.

Adsorption Site	Formation Energy (eV)	Adsorption Site	Formation Energy (eV)
**1**	−0.555	**7**	−0.169
**2**	−0.295	**8**	−0.167
**3**	−0.173	**9**	−0.402
**4**	−0.176	**10**	−0.353
**5**	−0.143	**11**	−0.514
**6**	−0.19	**12**	−0.514

## Data Availability

Not applicable.

## Supplementary Materials

The following supporting information can be downloaded at: https://www.mdpi.com/article/10.3390/ma16114041/s1, Figure S1: (a) Electronic band structure and (b) electron density of state of b-SiC_2_ for ferromagnetic orderings. The variation of (c) spin-down and (d) spin-up band gap of nanoribbons with width w. Figure S2: (a) Differential charge densities of armchair g-SiC3 nanoribbon. Yellow and blue colors indicate electron accumulation and depletion, respectively. Partial charge density distributions of the (b) valence band and (c) conduction band at Γ. Figure S3: The spin-polarized electron density of zigzag g-SiC3 nanoribbon for ferromagnetic orderings. Figure S4: Energy profile for Li diffusion in the g-SiC3 nanoribbons along paths (a) 6→5, (b) 6→8, (c) 8→9, and (d) 9→12.
