# Supplementary material for: Electronic Structures of Penta-SiC2 and g-SiC3 Nanoribbons: A First-Principles Study
Source: Materials (Basel). 2023 May 29;16(11):4041. doi: 10.3390/ma16114041 (PMC10254421; doi:10.3390/ma16114041)
Supplement: Supplementary file 1 [file materials-16-04041-s001.zip › materials-2374700-supplementary.pdf]

Supplementary Material for

Electronic Structures of Penta-SiC2 and g-SiC3 Nanoribbons: A First-Principles Study

Zhichao Liu [1], Xiaobiao Liu [2] and Junru Wang [1,*]

1) School of Physics and Electronic Informations, Yantai University, Yantai 264005, China
2) School of Sciences, Henan Agricultural University, Zhengzhou 450002, China

* Corresponding author. E-mail: wjr@ytu.edu.cn

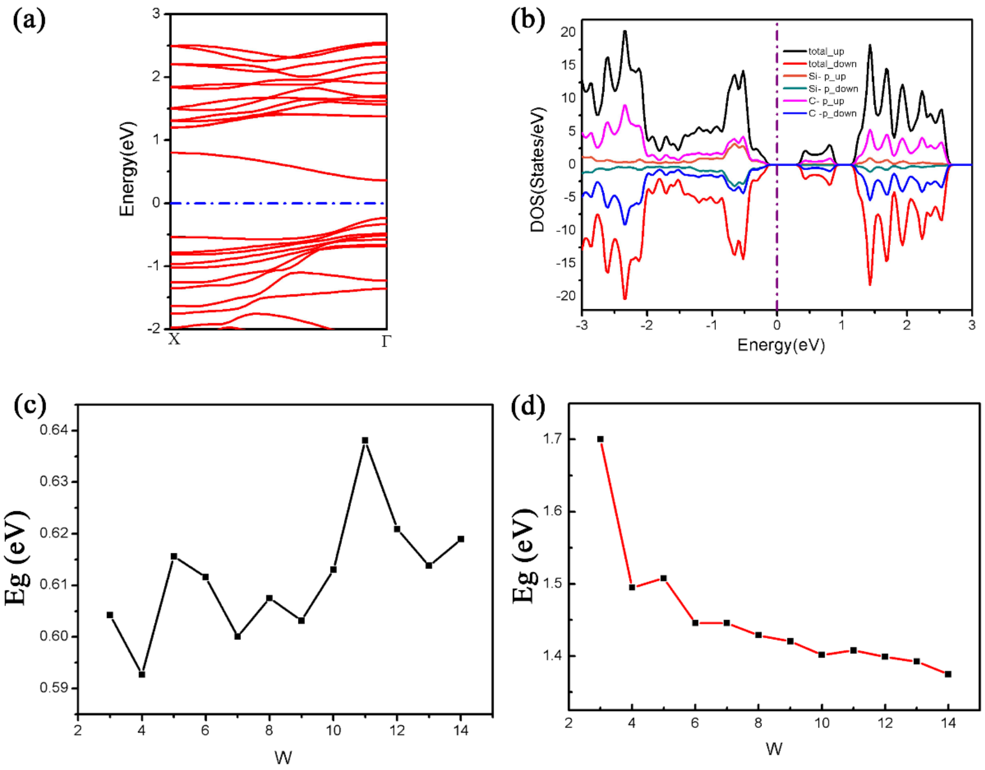

Figure S1. (a) Electronic band structure and (b) electron density of state of b-SiC_2 for ferromagnetic orderings. The variation of (c) spin-down and (d) spin-up band gap of nanoribbons with width w.

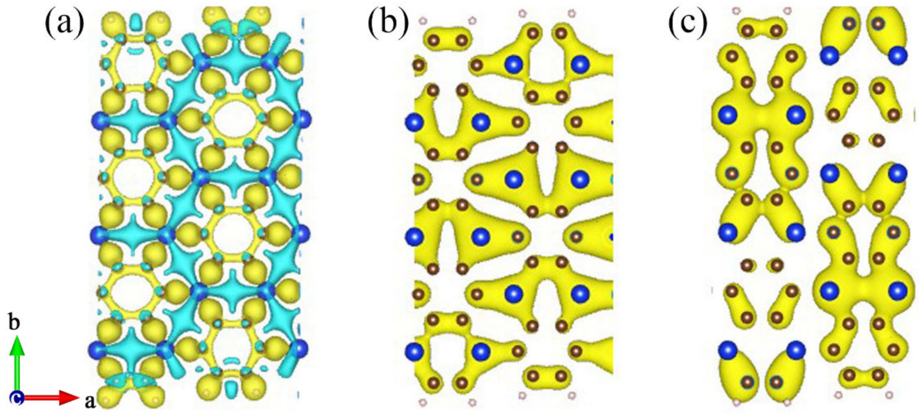

Figure S2. (a) Differential charge densities of armchair g-SiC_3 nanoribbon. Yellow and blue colors indicate electron accumulation and depletion, respectively. Partial charge density distributions of the (b) valence band and (c) conduction band at Γ.

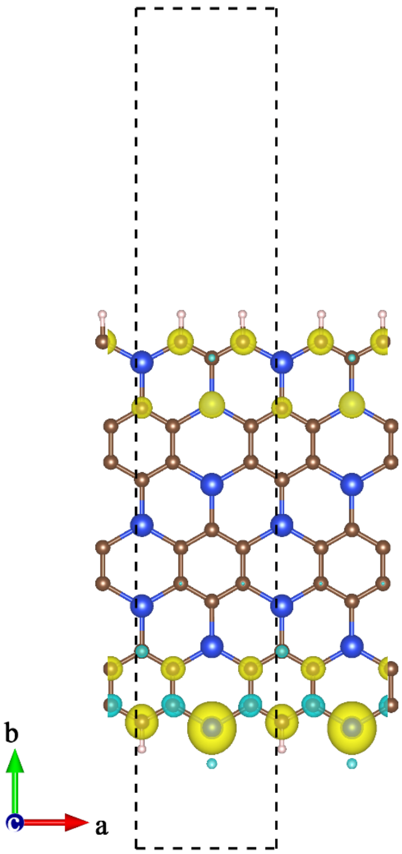

Figure S3. The spin-polarized electron density of zigzag g-SiC_3 nanoribbon for ferromagnetic orderings.

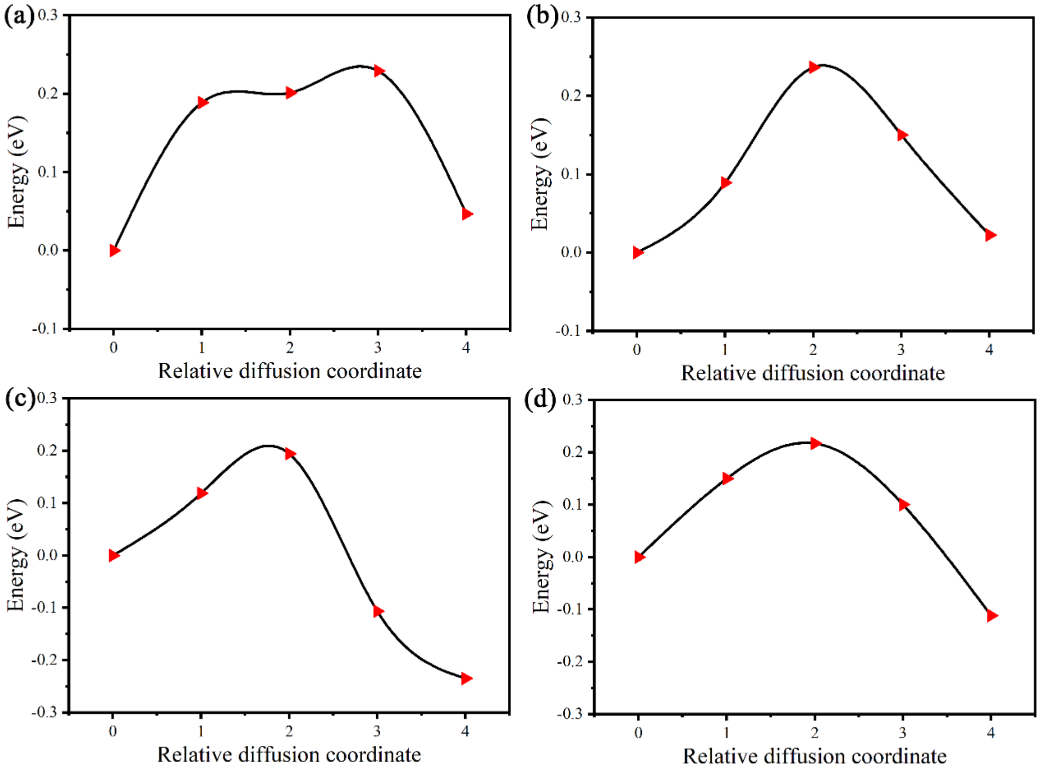

Figure S4. Energy profile for Li diffusion in the g-SiC_3 nanoribbons along paths (a) $6 \rightarrow 5$, (b) $6 \rightarrow 8$, (c) $8 \rightarrow 9$, and (d) $9 \rightarrow 12$.
